# Anti-Tumor Activity of a Novel HS-Mimetic-Vascular Endothelial Growth Factor Binding Small Molecule

**DOI:** 10.1371/journal.pone.0039444

**Published:** 2012-08-15

**Authors:** Basappa, Kazuyuki Sugahara, Kuntebommanahalli N. Thimmaiah, Hemant K. Bid, Peter J. Houghton, Kanchugarakoppal S. Rangappa

**Affiliations:** 1 Faculty of Advanced Life Sciences, Hokkaido University, Sapporo, Japan; 2 Chemical Biology and Therapeutics, St. Jude Children's Research Hospital, Memphis, Tennessee, United States of America; 3 Center for Childhood Cancer, Nationwide Children's Hospital, Columbus, Ohio, United States of America; 4 Department of Studies in Chemistry, University of Mysore, Mysore, India; 5 Singapore-MIT Alliance for Research and Technology, Singapore, Singapore; 6 Department of Chemistry, Bangalore University, Bangalore, India; Medical College of Wisconsin, United States of America

## Abstract

The angiogenic process is controlled by variety of factors of which the vascular endothelial growth factor (VEGF) pathway plays a major role. A series of heparan sulfate mimetic small molecules targeting VEGF/VEGFR pathway has been synthesized. Among them, **compound 8** (2-butyl-5-chloro-3-(4-nitro-benzyl)-3H-imidazole-4-carbaldehyde) was identified as a significant binding molecule for the heparin-binding domain of VEGF, determined by high-throughput-surface plasmon resonance assay. The data predicted strong binding of **compound 8** with VEGF which may prevent the binding of VEGF to its receptor. We compared the structure of **compound 8** with heparan sulfate (HS), which have in common the functional ionic groups such as sulfate, nitro and carbaldehyde that can be located in similar positions of the disaccharide structure of HS. Molecular docking studies predicted that **compound 8** binds at the heparin binding domain of VEGF through strong hydrogen bonding with Lys-30 and Gln-20 amino acid residues, and consistent with the prediction, **compound 8** inhibited binding of VEGF to immobilized heparin. *In vitro* studies showed that **compound 8** inhibits the VEGF-induced proliferation migration and tube formation of mouse vascular endothelial cells, and finally the invasion of a murine osteosarcoma cell line (LM8G7) which secrets high levels of VEGF. *In vivo*, these effects produce significant decrease of tumor burden in an experimental model of liver metastasis. Collectively, these data indicate that **compound 8** may prevent tumor growth through a direct effect on tumor cell proliferation and by inhibition of endothelial cell migration and angiogenesis mediated by VEGF. In conclusion, **compound 8** may normalize the tumor vasculature and microenvironment in tumors probably by inhibiting the binding of VEGF to its receptor.

## Introduction

There is a need for anticancer agents with novel mechanisms of action [1]. Recently identified molecular targets for new anticancer agents include, inducers of cell differentiation, cell cycle arrest, and apoptosis, as well as inhibitors of mitogenic signaling pathways elicited by growth factors and cytokines [2], [3], [4]. A largely unexplored opportunity is the use of carbohydrate mimetics as drugs [5]. The development of new synthetic methods for sugar mimetics has led to a wide variety of novel structures, such as oxazines, triazole-imines, isoxazolines, and imidazole derivatives [6], [7], [8] that may exert anti-tumor activity. The cell surface and extracellular matrix heparan sulfate (HS) is known to play a major role in tumor metastasis, and acts as a storage site for various proteins [9]. Heparan sulfate glycosaminoglycans (HSGAGs) have been found to play regulatory roles in many biological functions; both normal physiological processes (e.g., embryogenesis) as well as pathological processes (e.g., tumorigenesis and metastasis).

The approach of modulating tumor progression with drugs that inhibit growth of tumors/endothelial cells or the activities of angiostatic factors represents a new concept in adjuvant chemotherapy when used alone or in combination with classic antitumor drugs. This approach is being explored either with the use of monoclonal antibodies directed to the factors [10] or with growth factor-complexing molecules of different origin such as suramin [11], polysaccharide [12], and anionic compounds [13]. The sulphonated derivative of distamycin A, FCE 26644, has been found to inhibit angiogenesis and showed anti-tumor activity by complexing with the growth factors [14], [15]. Most recently, a VEGF-A receptor neuropilin 1 binding ligand (EG00229) was designed and synthesized that reduced the viability of A549 lung carcinoma cells [16]. The proliferation and growth of solid tumors *in vivo* is dependent on various growth factors and cytokines, either through a direct stimulus to cell division and/or through activation of neovascularization, an essential event in tumor progression and metastasis [17]. The increased production of vascular endothelial growth factor (VEGF), fibroblast growth factor-2 (FGF-2), heparin-binding epidermal growth factor-like growth factor (HB-EGF), and tumor necrosis factor-α (TNF-α), as well as the overexpression of their receptors has been reported in a variety of human tumors [17]. It is well known that both VEGF and FGF-2 are capable of stimulating angiogenesis *in vitro* and *in vivo* [18]. During angiogenesis, the proliferation, migration and remodelling of fully differentiated endothelial cells to form microvessels are regulated by growth factors such as VEGF or FGF secreted by tumor cells [19]. Therefore, the inhibition of VEGF or FGF expression or blocking their mitogenic response on endothelial cells might have therapeutic value in the treatment of a variety of cancers [20].

The enormous structural diversity of HSGAGs makes it possible for them to interact specifically with a wide variety of proteins and other ligands. Synthesis of such HS mimetic compounds with antitumor and antiangiogenic properties are currently an active area of research. In order to dissect a variety of the pathological roles of HSGAGs and specifically to identify and develop novel anti-tumor agents, we explored virtual libraries of HSGAG-mimetic compounds using database search techniques. The literature revealed the anti-tumor and anti-heparanase activities of a non-sugar-based HS mimetic compound 2-[3-nitro-4-(phenylthio)benzoyl]benzoic acid (KI-105) [21]. We herein, synthesized these types of compounds, which bear HS mimetic structure in order to evaluate their efficacy in preventing the invasion and proliferation of tumor cells. Here, we prepared the hybrid of HS-mimetic core structure of KI-105 by replacing the benzene group by imidazole, since the chemistry of imidazole occupies an extremely important niche within the family of 5-membered heterocyclic compounds. 2-butyl-5-chloro-3-(4-nitro-benzyl)-3H-imidazole-4-carbaldehyde (**compound 8**) is considered to mimic the HS non-structurally. We report that the **compound 8**, directly binds to the heparin binding domain of VEGF, as detected by high throughput surface plasmon resonance (SPR) analysis, as well as by *in silico* binding analysis, and showed promising antitumor activity in experimental model of liver matastasis.

## Results and Discussion

Several studies have shown that HS mimetics act as antitumor agents [21]. However, the pleiotropic effects and interactions of such mimetics with heparin-binding proteins might elicit off-target effects associated with toxicity. Low molecular weight HS mimetics which perform multiple biological functions *in vivo* and *in vitro* with high specificity are rare. To this end, we sought to synthesize a series of novel non-sugar-based compounds which can mimic the HS non-structurally.

### Synthesis

**Figure 1:**
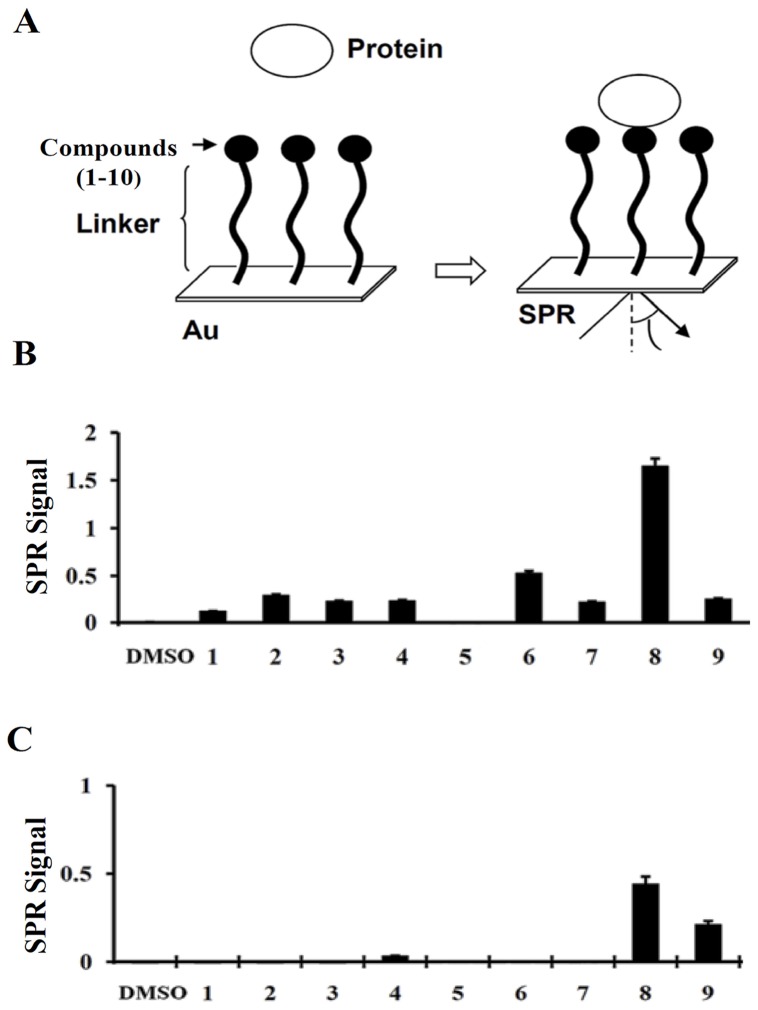
Reagents and condition: a) R_1_-CHO, K_2_CO_3_, Methanol, RT; b) 2,6-difluorobenzaldehyde, Conc. H_2_SO_4_, ethanol, reflux; c) R_1_-Cl, K_2_CO_3_, DMF, RT.

Synthesis of small molecules (**1–9**) is shown in **Figure 1**, which depicts the synthesis of 1,3-oxazine derivatives, 1,2,4-triazole derivatives, and imidazole derivatives. 1,3-oxazine derivatives **1–4**, were prepared by the cyclization of 1-[2-amino-1-(4-methoxy-phenyl)-ethyl]-cyclohexanol monoacetate with aromatic or aliphatic aldehydes in the presence of potassium carbonate [22], [23]. 4-Amino-4,5-disubstituted-[1,2,4] triazole-3-thiols **5–7**, were synthesized by condensation reaction of 4-amino-5-methy/ethyl/phenyl-4H-[1,2,4]triazole-3-thiols with 1,6-difluorobenzaldehyde in presence of catalytic amount of concentrated sulphuric acid in ethanolic media (**Figure 1**). Microwave-assisted synthesis of N-substituted 2-butyl-5-chloro-3H-imidazole-4-carbaldehyde derivatives **8** and **9**, were synthesized as reported [24], [25].

### High throughput surface plasmon resonance (SPR) screening of small molecules binding to growth factors

**Figure 2:**
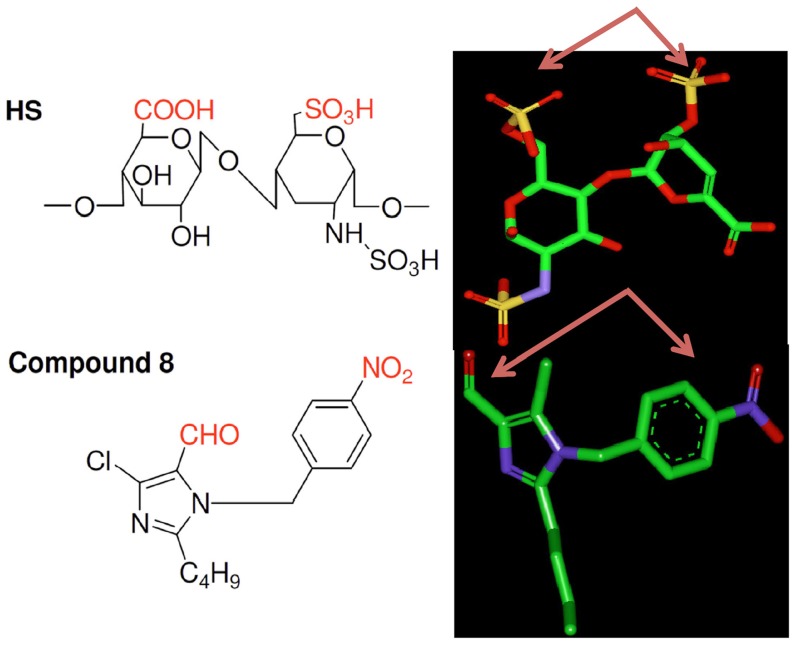
Interaction between the small molecules and growth factors. An overview of the SPR analysis showing the interaction between the sugar mimetics and growth factors/cytokines (A). Small molecules (**1–9**) were immobilized on the photoaffinity-linker-coated gold substrates. Interactions were detected between the small molecules (**1–9**) and the growth factors in solution by SPR imaging as described under “Experimental Procedures”. The maximum SPR signal strength observed between the small molecules **(1–9**) and VEGF (B), or FGF-2 (C) are presented.

We immobilized the HS-mimetic small molecule libraries (around 60) in order to determine their binding ability with heparin-binding growth factors such as VEGF, FGF-2, TNF-α, midkine, pleotrophin, or HB-EGF by conducting a novel SPR assay (Basappa et al, Cancer Letter, 2010; Supplementary Fig. 1–3). An overview of the SPR analysis is shown in **Figure 2A**. Compounds were immobilized on the photoaffinity-linker-coated gold substrates (PGSs) as reported previously [26], [27]. In our *in vitro* experimental conditions, strong SPR signals for the direct binding of the selected compounds (**1–9**) with VEGF (**Figure 2B**) or FGF-2 (**Figure 2C**) were found. Compound **2** bound to VEGF moderately when compared to other oxazine molecules tested during the assay. Among the triazole compounds like **5**, **6**, and **7**, compound **6** bound to VEGF significantly. More importantly, the imidazole derivative, **compound 8**, bound to VEGF very strongly, when compared to other screened molecules by SPR assay. It also interacted with FGF-2 significantly, although with weaker affinity compared to its VEGF binding. Some of the synthesized compounds weakly bound to other heparin-binding growth factors such as HB-EGF or TNF-α (data not shown). In addition, none of the compounds showed any binding to pleiotrophin and midkine (data not shown). The assay was found to be specific in terms of binding; hence these results indicate the binding specificity of **compound 8** towards VEGF and led us to speculate that this molecule may be used to modulate the cellular processes that are mediated by VEGF.

### Molecular interaction of compound 8 with the heparin binding site of VEGF_165_

**Figure 3:**
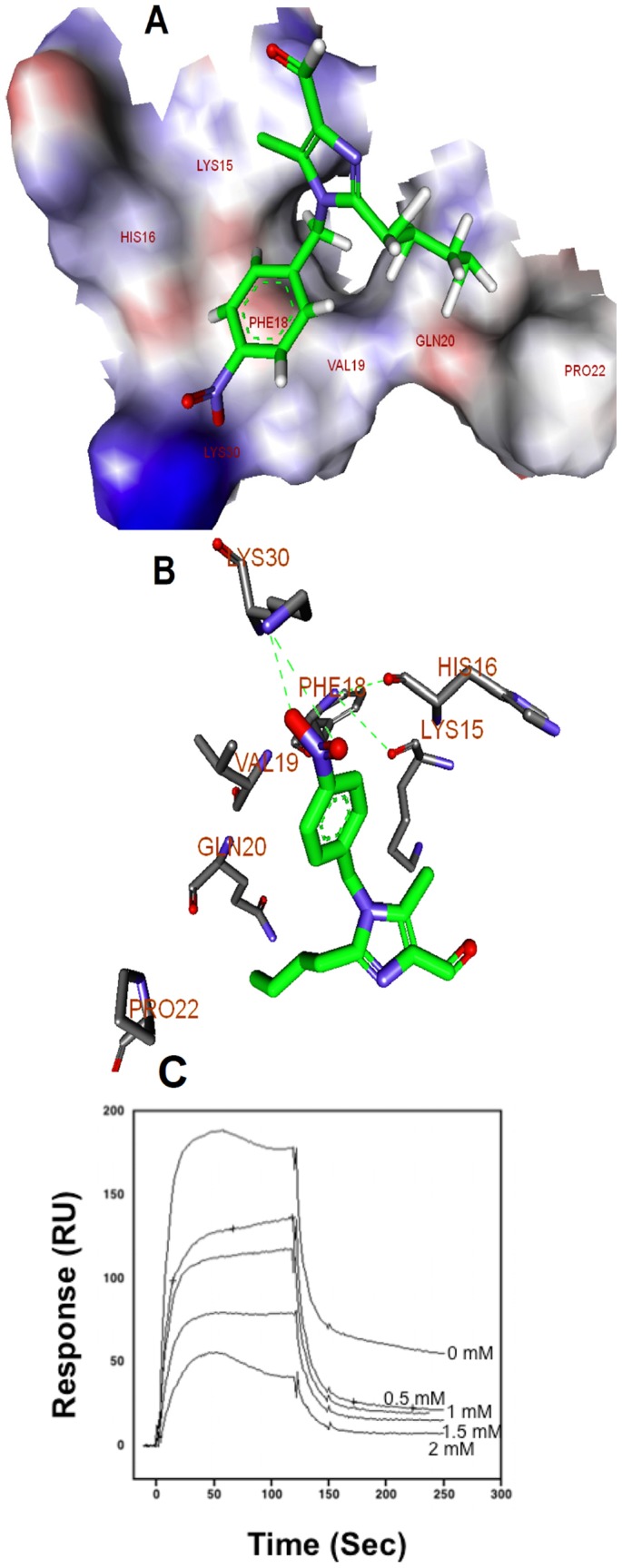
Discovery of New HS mimetic small molecule. *Left*. Chemical structure of a HS disaccharide unit (1), HexUA(2S)-GlcNAc(6S), and **compound 8** are also shown. *Right*. Energy-minimized structures of HS and **compound 8**. The arrows show the anionic functional groups. The carbon, oxygen, nitrogen, and sulfur atoms are represented in green, red, blue, and yellow, respectively. The hydrogen atoms are not shown.

**Figure 4:**
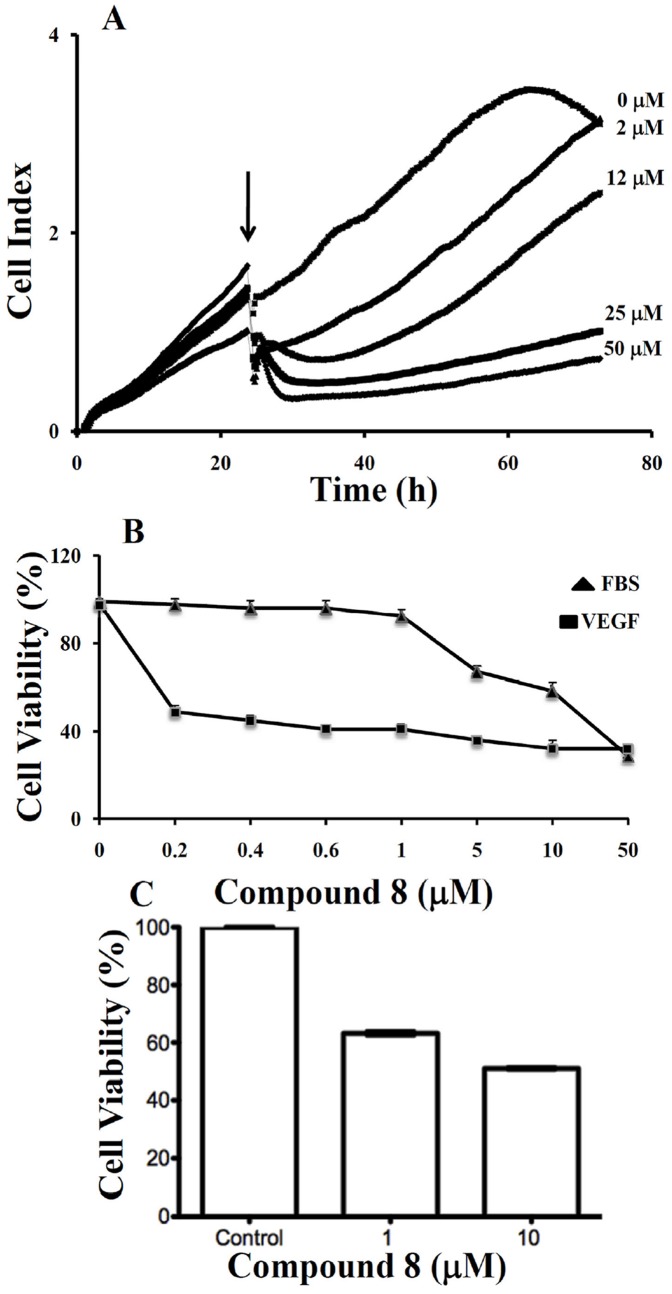
Molecular basis for the interaction between compound 8 and heparin binding domain of VEGF_165_. A. Binding mode of **compound 8** with heparin binding site of VEGF_165_. The amino acid residues of heparin binding site of VEGF_165_ are shown in stick models. B. Interactions of the **compound 8** within the heparin binding pocket of VEGF_165_ with putative hydrogen bonds shown as green dotted lines. The **compound 8** is shown in green color. The bonding between the **compound 8** and the heparin binding domain of VEGF_165_ are shown in yellow color. All the hydrogen atoms are not shown. The picture is rendered in Discovery Studio, version 2.5 (right panel). C. Binding of VEGF_165_ to immobilised-heparin. Various concentrations of **compound 8** and VEGF_165_ (250 nM) mixture in running buffer was injected onto the surface of the heparin-immobilised sensor chip. Sensograms obtained were overlaid using a BIA evaluation software (version 3.1).

We initially prepared the energy minimized structures of the synthesized molecules. The results of the calculations were compared those for HexUA-GlcNAc(6S). Finally, we selected **compound 8** as the core structure (Figure 3A). The energy-minimized structures of HexUA(2S)-GlcNAc(6S) and **compound 8** revealed that anionic groups, such as sulfate, carboxylic acid, carbaldehyde and nitro functional groups, are able to locate in similar positions and directions (Figure 3B). Because of structural similarity (by functional groups) to HS, **compound 8** could bind to VEGF_165_ in the heparin binding site. **Compound 8** interacts with VEGF strongly, and FGF-2 weakly as determined by SPR assay, hence we performed the molecular docking processes using CDOCKER, which docks ligands to the heparin binding site of VEGF (27). The molecular docking studies predicted that **compound 8** binds to the heparin binding domain of VEGF (Figure 4A) with high binding affinity while some other compounds do not show affinity towards the heparin binding site of VEGF (data not shown). **Compound 8** showed the CDOCKER score (-CDOCKER ENERGY) of 24.7, a high value, which indicates more favourable binding. This score includes internal ligand strain energy and receptor-ligand interaction energy, and is used to sort the different conformations of each input ligand. Visual analysis of the docked **compound 8** shows the imidazole and benzene nucleus resides in the pocket formed by Val-19, Phe-18, His-15, Lys-16, and Pro-9. In addition, the **compound 8** bound through hydrogen bonding with Lys-30 and Gln-20 residues at the heparin binding pocket of VEGF (**Figure 4B**). On the other hand, we found the hydrophobic interaction of the butyl group of **compound 8** with the heparin binding domain of FGF-2 with a CDOCKER score of 4.8, which is lower when compared to VEGF interaction CDOCKER score (Fig. S1). This revealed that the binding of **compound 8** with VEGF is stronger when compared to FGF-2. A similar observation has been reported previously for the compound KI-105 that bound to the HS binding site of heparanase [21]. We know that HS binds to heparin-binding growth factors and cytokines such as VEGF and FGF-2. We analysed the interaction between VEGF_165_ and bound heparin using the BIAcore system. This study revealed that **compound 8** inhibited the binding of VEGF_165_ to the immobilised heparin (**Figure 4C**). Although the concentration of **compound 8** needed to inhibit the binding of VEGF to the heparin was high, these data show that the molecular interaction takes place. In addition, the structurally related compound 7 that does not bind VEGF, failed to inhibit the binding of VEGF to heparin, suggesting selectivity. The results indicate that **compound 8** initially competes or is bound initially to the heparin binding domain of VEGF and thereby decreases the binding of VEGF to the immobilized heparin.

### Effect of small molecules on the proliferation of tumor cells

Small-molecules that intervene in biological systems have been sought as tools to perturb signaling pathways and of course as therapeutic agents for disease. Heparin and HS structural mimetics have been reported as antitumor agents that significantly inhibit tumor growth, metastasis, and angiogenesis. To validate the binding ability of these compounds with VEGF and to further elucidate the biological functions of these molecules, we initially examined the effects of compounds 1–9 on the proliferation of murine osteosarcoma cell lines, LM8 and LM8G7, which are known to the secrete proangiogenic factor VEGF [28]. Compounds **1–4, 6, 7**, **and 9** did not show promising inhibitory activity towards the proliferation of osteosarcoma cancer cells (**Table 1**). Compounds **5** and **6** inhibited moderately the proliferation of LM8 and LM8G7 cells. In contrast to other compounds, **compound 8**, which bears an imidazole moiety, showed more potent inhibition towards the proliferation of VEGF-secreting LM8 and LM8G7 cells with an IC_50_ values of 7 µM and 4.5 µM, respectively. In comparison cisplatin was less potent with IC_50_ concentrations of 30 and 15 µM, respectively (Table 1). The inhibitory activity of suramin was comparable with the inhibitory activity of **compound 8** on the proliferation of tumor cells.

**Table 1:** Inhibition of the proliferation of tumor and endothelial cells by compounds 1–9 (IC_50_ in µM).

Compound	R1/R2/R3	LM8	LM8G7	UV♀2
**1**	H	NS^a^	NS	NS
**2**	4-Fluoro-phenyl	64±0.5	86±0.1	NS
**3**	4-Chloro-phenyl	NS	>100	NS
**4**	pyridin-3-yl	95±6.7	NS	>100
**5**	Methyl	36±6.4	NS	45±2.1
**6**	Ethyl	64±6.7	23±0.5	14±4.6
**7**	Phenyl	>100	NS	NS
**8**	4-Nitro-phenyl	6.8±0.3	5±2.8	42±0.1
**9**	6-methyl-benzo[1,3] dioxol-5-yl methyl)-	46±6.7	NS	NS
**Suramin**		12±2.8	14±24	34±1.4
**Cisplatin**		30±24	15±0.6	12±0.4

a NS, not significant.

### Real-time monitoring of the effect of compound 8 on the proliferation of LM8G7 cells

**Figure 5:**
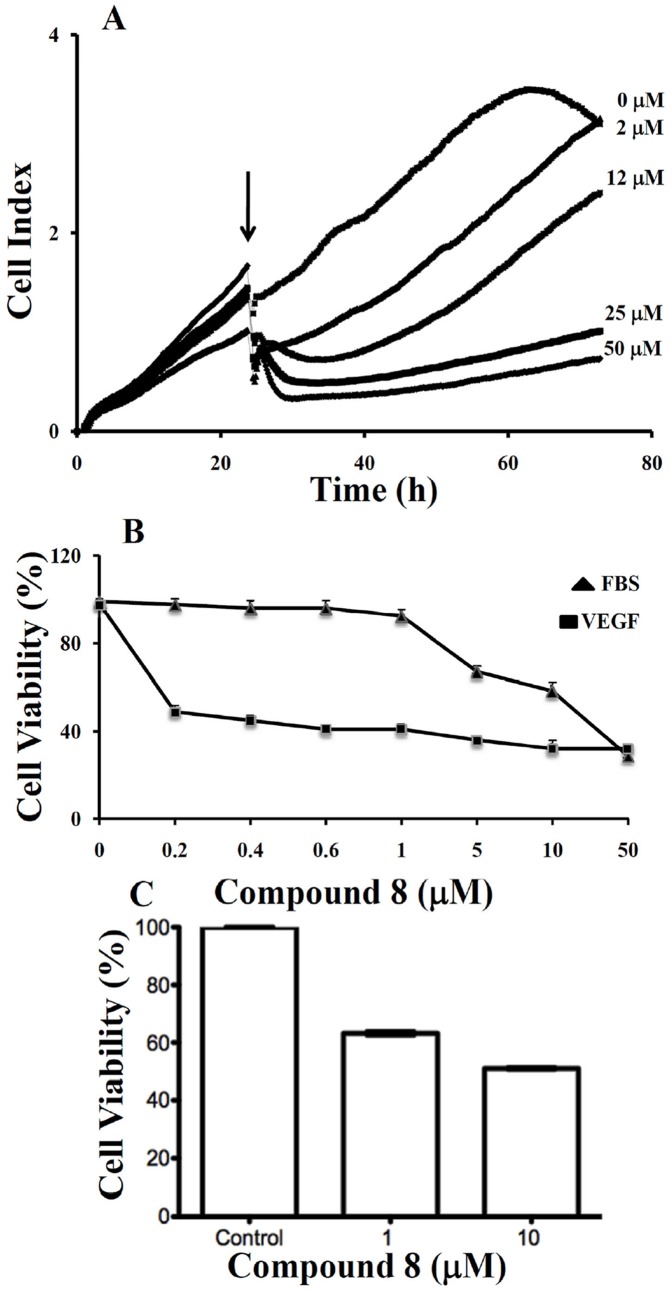
A. Real-time monitoring of the effects of compound 8 on the proliferation of LM8G7 cells. Cells were seeded in ACEA's 96× e-plate™ at a density of 5×10^3^ cells per well, and continuously monitored using the RT-CES system up to 24 h, at which point Compound 8 (2 to 50 µM) was added. The cell index is plotted against time. The arrow indicates the time of the addition of **compound 8**. Data represent the mean values ± S.D. for three identical wells from three independent experiments. **B**, **compound 8** poorly inhibited proliferation of UV♀2 under serum-replete conditions (FBS), but potently inhibited VEGF-dependent proliferation of UV♀2 cells as analysed by TetraColor One assay. Results were normalised to DMSO controls. Data represent mean values ± SD for three independent experiments. ^*^ *P*<0.05 versus control. ^**^ *P*<0.01 versus control. **C**, **compound 8** inhibits VEGF-stimulated proliferation of human vascular endothelial cells. HUVEC cells were seeded on 6-well plates at a density of approximately 1×10^5^ cells/well in M200 medium supplement with LSGS (Low serum growth Supplement). The next day cells were stimulated with 10 ng/mL of VEGF in the presence or absence of 1 µM and 10 µM **compound 8**. After 48 hrs, Alamar Blue was added directly into culture media at a final concentration of 10% and the plate was returned to the incubator. Optical density (OD) of the plate was measured at 540 and 630 nm. As a negative control, AB was added to medium without cells.

Elimination or arrest of tumor cell proliferation in the target organ are the eventual objectives of anticancer therapy. We monitored the effect of **compound 8** (2 to 50 µM) on the proliferation of VEGF secreting LM8G7 cells using real-time cell electronic sensing system™ (RT-CES) to confirm the results obtained through TetraColor One assay. **Compound 8** inhibited the proliferation of LM8G7 cells in a concentration-dependent manner with an IC_50_ value 5 µM, confirming its anti-proliferative effect on LM8G7 cells (**Figure 5A**).

### Effect of small molecules on the proliferation of endothelial cells

The proliferation and migration of endothelial cells to form microvessels is critical for angiogenesis, and inhibition of angiogenic factor-mediated proliferation of endothelial cells has been shown to be an effective antiangiogenic therapy [29]. Hence, we tested the effects of these compounds on the VEGF-induced proliferation of endothelial (UV♀2) cells. Except for compounds **6**, the remaining compounds had no significant effects on the proliferation of UV♀2 cells, whereas compound **6** which had weak binding with VEGF moderately inhibited its proliferation. **Compound 8**, which strongly binds to VEGF, markedly inhibited the proliferation of UV♀2 cells with an IC_50_ value of 42 µM (Table 1). These results show that **compound 8** suppressed the proliferation of endothelial cells, but the concentration of **compound 8** required to suppress cell proliferation was high compared with that required to suppress proliferation of LM8G7 osteosarcoma cells.

In contrast to UV♀2 cells treated with **compound 8** under serum-containing conditions (10%FBS), **compound 8** showed a potent inhibitory effect on the VEGF-stimulated proliferation with an IC_50_ value 0.3 µM (**Figure 5B**). These results indicate that **compound 8** inhibited endothelial cell proliferation through inhibition of VEGF receptor function.

We next examined the effect of **compound 8** to suppress VEGF-stimulated proliferation of HUVECs. As shown in **Figure 5C**, proliferation was inhibited approximately 40% at 1 µM, and by 50% at 10 µM. Taken together, these data suggest that **compound 8** can block the VEGF-mediated angiogenic response in endothelial cells.

### Compound 8 suppresses VEGF-induced migration and tube formation of endothelial cells

**Figure 6:**
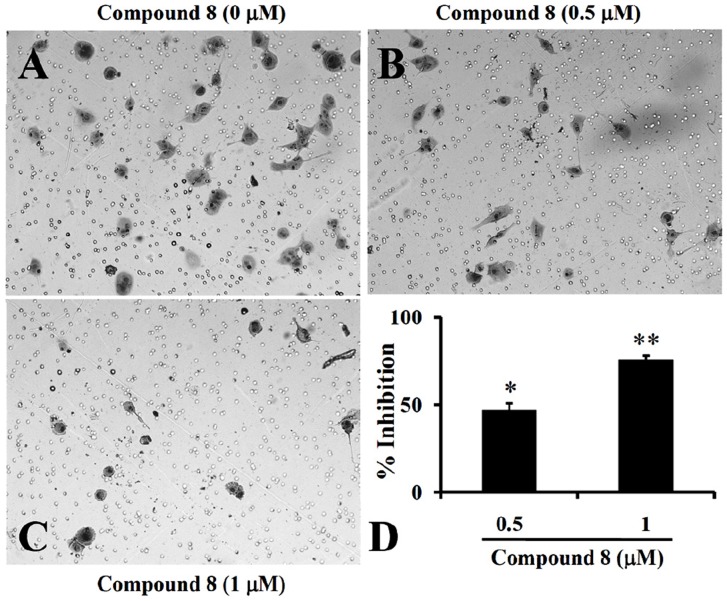
Compound 8 suppresses VEGF-induced migration and angiogenesis. UV♀2 cells (2.5×10^4^) were seeded along with VEGF (2 ng/ml) in Boyden-chambers and incubated for 24 h with DMEM (**A**) or a medium containing 0.5 µM of **compound 8** (**B**) or 1 µM **compound 8** (**C**); the photographs (100×) shows the cell on the lower surface of the filter (migrated) stained with the Diff-Quick solution. (**D**) Inhibition rate of the **compound 8** on UV♀2 migration was presented as described under “Experimental Procedures”. Data represent the mean values ± S.D. for three identical wells from three independent experiments. Bars represent the mean ± SD of three independent experiments. * *P*<0.05 versus control. ** *P*<0.01 versus control. The UV♀2 cells were seeded in 6-well plates pre-coated with Matrigel™ (ECM 625, Chemicon) was added to a 24-well plate in a final volume of 100 µL and allowed to solidify at 37°C for 30 min. UV♀2 cells (4×10^5^ cells) were seeded to the ECMatrix™-coated wells in the presence or absence of **compound 8** (0.5 (F) or 1 µM (G) along with 2 ng/ml VEGF. After 18 h of culture, the reorganization of the sub-confluent monolayer of UV♀2 cells in 3-dimensional Matrigel™ was monitored and photographed under an Olympus FX380 microscope attached to a 3CCD camera.

Endothelial cell migration is critical events for angiogenesis. To examine whether **compound 8** inhibited migration of UV♀2 endothelial cells the effect of **compound 8** against the VEGF-induced migration across the porous membrane of BD BioCoat™ chambers was determined. VEGF significantly enhanced migratory activity and cotreatment with **compound 8** resulted in significant inhibition of VEGF-induced migration. Cells were treated with VEGF, drug vehicle (0.5% DMSO, Control) or **compound 8** at 0.5 and 1.0 µM. Representative photomicrographs are shown in **Figure 6A–C**. Quantification of the reduction in migration showed that **compound 8** significantly inhibited VEGF-induced migration of endothelial cells by 52, and 83%, at 0.5 and 1.0 µM, respectively (**Figure 6D**). Further, **compound 8** at 1 µM was non-toxic as is evident from the TetraColor One assay (Table 1); hence, the inhibitory effect could not be attributed to cytotoxic activity.

Stimulation of endothelial cells on ECMatrix™ by angiogenic factors such as VEGF promotes differentiation to form capillary-like tubes. We performed the tube formation assay to assess any specific effect of **compound 8** on this process. The results showed that VEGF significantly increased tube formation (**Figure 6E**) and cotreatment of endothelial cells with **compound 8** at 0.5 to 1 µM resulted in strong inhibition of VEGF-induced tube formation in a concentration-dependent manner (**Figure 6F and G**). At 1 µM **compound 8** resulted in complete inhibition of the tube formation by the endothelial cells. The combined results show that **compound 8** suppressed the proliferation of endothelial cells, but the concentration of **compound 8** required to suppress the proliferation was high compared with that required to suppress the tube formation and cell migration. However, the compound **8** failed to inhibit the FGF-2-induced tube formation by the endothelial cells at the tested concentrations (**Figure S2**). Taken together, we observed that **compound 8** may block the VEGF-mediated angiogenic response in endothelial cells.

### Effect of compound 8 on the invasion of LM8G7 cells

**Figure 7:**
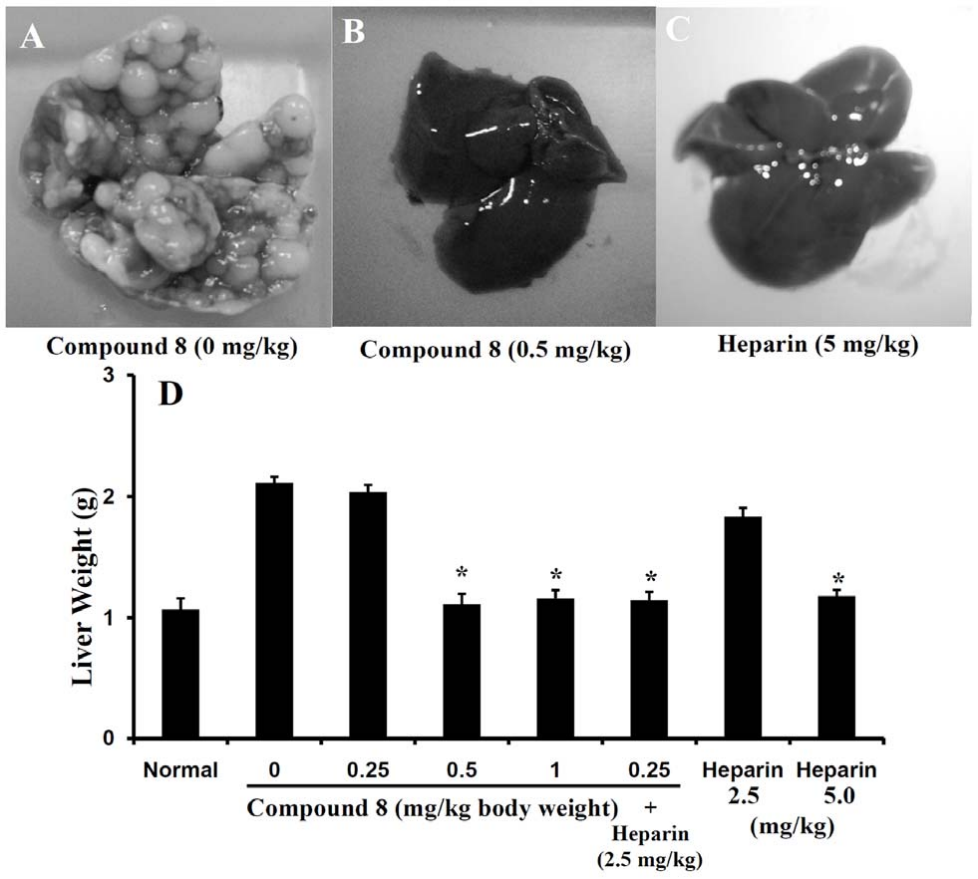
Effect of compound 8 on the invasion of LM8G7 cells. Cells (2.5×10^4^) were seeded in Matrigel™-coated Boyden chambers and were incubated for 24 h with DMEM (A) or a medium containing 0.5 µM of **compound 8** (B) or 1 µM **compound 8** (C); the photographs (100×) shows the cell on the lower surface of the filter (invaded) stained with the Diff-Quick solution. (D) Inhibition rate of the **compound 8** on LM8G7 invasion was presented as described under “Experimental Procedures”. Bars represent the mean ± SD of three independent experiments. * *P*<0.05 versus control. ** *P*<0.01 versus control.

The effect of **compound 8** on the invasion of highly metastatic LM8G7 cells across Matrigel™-coated porous membranes was studied. Control (DMSO-treated) LM8G7 cells were highly invasive in the assay (**Figure 7A**). **Compound 8** at 0.5 and 1 µM (**Figure 7B and C**) effectively inhibited the invasion of LM8G7 cells by 46, and 75% (**Figure 7D**), respectively, when compared to control. Compounds **1–7** and **9** failed to inhibit the invasion of LM8G7 cells at 1 µM concentration effectively (data not shown).

### Compound 8 prevents experimental liver metastasis

**Figure 8:**
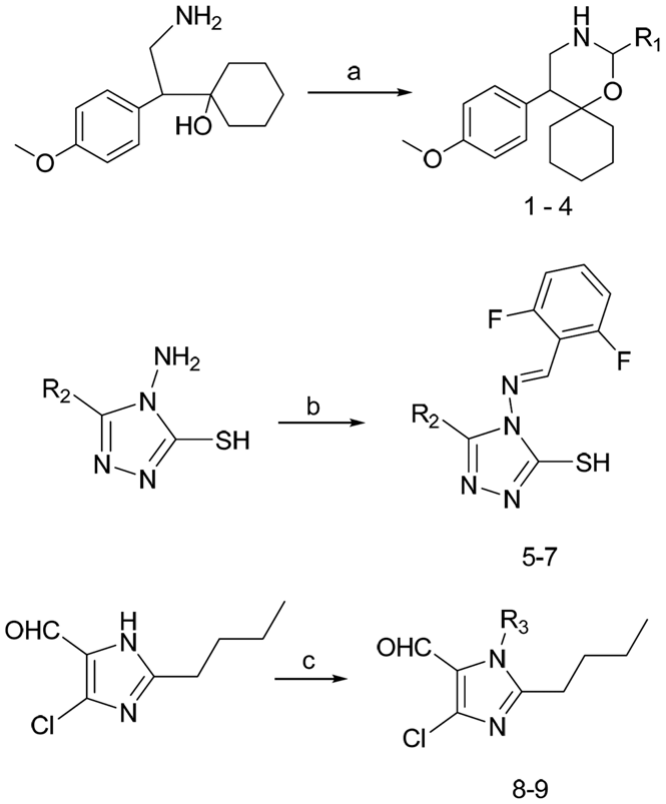
Effect of the compound 8 against the metastatic potential of LM8G7 cells. C3H/HeN mice were intravenously injected with LM8G7 cells via tail. Some mice received an intravenous injection of **compound 8** (0.25, 0.5, or 1.0 mg/kg) on day 3, 5 and 10. After 4 weeks, the mice were sacrificed, the number of liver nodules was counted macroscopically, and the liver weight was measured in the control and **compound 8**-treated animals. Representative livers from mice injected with LM8G7 cells treated with DMEM (A), **compound 8** (0.5 mg/kg) (B), and heparin (5 mg/kg) (C) are shown. The average liver weight of the mock and the **compound 8** treated mice (D). Heparin from porcine intestinal mucosa was used as a positive control. The possible synergistic or additive effects of **compound 8** and heparin were investigated by subsequent administration of **compound 8** (0.25 mg/kg) and heparin (2.5 mg/kg) as described above. Data represent mean values ± SD for three independent experiments and each experiment was conducted with 6 mice per group. ^*^ *P*<0.05 versus control. Mann-Whitney *U* test.

Because **compound 8** potently inhibited endothelial cell migration and tube formation, as well as LM8G7 cell proliferation, we examined the *in vivo* anti-tumor effect of **compound 8**. Following intravenous injection of LM8G7 cells mice developed metastatic nodules in the liver within 30 days (**Figure 8A**). In contrast, mice treated intravenously with **compound 8** (0.5 or 1.0 mg/kg, days 3, 5, and 10) or heparin (5 mg/kg, used on the same schedule of administraion as a positive control) were completely free of metastatic nodules in the liver, **Figure 8B and C**, respectively. **Compound 8** at 0.25 mg/kg treatment, failed to inhibit the tumor formation by the LM8G7 cells (**Figure 8D**). The animals tolerated the dosages of **compound 8** (0.5 to 1.0 mg/kg), with no signs of toxicity or weight loss during the experiments (data not shown). Unlike heparin, **compound 8** did not show anti-coagulant activity *in vitro* (data not shown). In addition, heparin at 2.5 mg/kg dose failed to inhibit the metastasis of LM8G7 cells (**Figure 8D**). We also evaluated the effect of **compound 8** (0.25 mg/kg) in combination with heparin (2.5 mg/kg). As shown in **Figure 8D**, the combination treatment resulted in complete abrogation of metastatic tumor nodules.

### Summary

We demonstrated here that the **compound 8** strongly interacted with VEGF as evidenced by a novel SPR assay. Molecular docking calculations revealed that hydrogen bonding interactions may play crucial role in demonstrating biological activity. Concerning the mechanism of action, we observed that **compound 8** may displace VEGF from its specific binding sites on HUVECs. It is noteworthy that HS mimetic **compound 8** might therefore behave as an antagonist or partial agonist. Since heparin can interfere with VEGF action either by binding to a specific domain on VEGF or by interacting with its cellular binding sites, KDR or NRP1, Consequently, **compound 8** might affect both the ligand and its cellular binding sites. We show here that **compound 8** interacts directly with VEGF. This is in agreement with our previous findings that compound **v** bound to FGF2, and thus altering the conformation of ligand receptor complexes. **Compound 8** showed *in vitro* antiproliferative activity against LM8G7 osteosarcoma cells (endogenously expressing VEGF), and inhibited their invasiveness. **Compound 8** inhibited the VEGF induced endothelial tube formation by UV♀2 cells and suppressed VEGF-stimulated proliferation and tube formation of HUVEC cells. *In vivo*, **compound 8** potently inhibited formation of liver metastases following intravenous injection of LM8G7 osteosarcoma cell. Collectively, these data indicate that **compound 8** may prevent tumor growth through a direct effect on tumor cells by blocking VEGF paracrine or autocrine stimulated proliferation and by inhibition of endothelial cell migration and angiogenesis mediated by VEGF. In conclusion, the effects of **compound 8** are probably through inhibiting the binding of VEGF to its receptor. Furthermore, to the best of our knowledge, this is the first report on a non-structural HS mimetic, which interacts directly with VEGF. Our data suggest that **compound 8** has potential use to build a therapeutic drug for minimizing tumor growth and angiogenesis in VEGF related models.

## Materials and Methods

Cisplatin, recombinant human (rh)-FGF-2, and rh-VEGF_165_ were purchased from Wako Pure Chemicals Co. (Osaka, Japan). 100× non-essential amino acids, b-mercaptoethanol, 100× sodium pyruvate and L-glutamine were from GIBCO (Auckland, New Zealand). The cell proliferation assay kit Tetracolor One was obtained from Seikagaku Corp. (Tokyo, Japan) and the Diff-Quick solution was from International Reagent Corp. (Kobe, Japan). All other chemicals and reagents used were of the highest commercial grade available.

The melting points were determined on a SELACO-650 hot stage apparatus and are uncorrected. IR (KBr) spectra were recorded on a Jasco FT/IR-4100 Fourier transform infrared spectrometer, ^1^H NMR were recorded on a Shimadzu AMX 400 spectrometer using CDCl_3_ as solvent and TMS as an internal standard (chemical shift in ppm). Elemental analyses were obtained on a vario-EL instrument and were within ±0.4% of calculated values. Thin layer chromatography (TLC) was conducted on 0.25 mm silica gel plates (60F_254_, Merck). Visualization was made with ultraviolet light. All extracted solvents were dried over anhydrous Na_2_SO_4_ and evaporated with a BUCHI rotary evaporator. Silica gel (200–400 mesh) from Aldrich, Inc., was used for column chromatography. All other chemicals were obtained from Aldrich, Inc.

### Preparation of the compounds 1–4, 8, and 9

Compounds **1**, **8, and 9** were prepared by the methods described in our previous papers [25], [30].

### The compounds 5 to 7 were prepared by the following procedure

Equimolar amount of 4-amino-5-substituted-4H-[1,2,4]triazole-3-thiols and 2,6-difluoro-benzaldehyde were heated in ethanol (10 ml) until a clear solution was obtained. Then a few drops of conc. H_2_SO_4_ were added and the solution was refluxed for 3–4 h on a water bath. After completion of the reaction, the solvent was evaporated, water was added, and cooled to 10–15°C. The product was filtered at the same temperature, and the residue washed with chilled n-hexane, dried and recrystallised with ethyl acetate to obtain the pure compounds.

### 4-[(2,6-Difluoro-benzylidene)-amino]-5-methyl-4H-[1,2,4]triazole-3-thiol 5

The product was obtained from 4-amino-3-methyl-4*H*-[1,2,4]triazole-5-thiol (1 g, 7.6 mmol) and 2,6-difluoro-benzaldehyde (1.09 g, 8.3 mmol) as white crystalline solid (0.94 g, 94%). mp: 225–228°C. IR ν_max_ (KBr): 1625 (s), 1285 (m), 892 cm^−1^. ^1^H NMR (CDCl_3_ 400 MHz) δ: 13.8 (s, 1H, S-H), 10.6 (s, 1H, CH = N-), 7.65–7.74 (m, 2H, Ar-H), 7.27–7.33(t, 1H, Ar-H), 2.32 (s, 3H, CH_3_). Anal. (C_11_H_9_F_2_N_3_S) C, H, N.

### 4-[(2,6-Difluoro-benzylidene)-amino]-5-ethyl-4H-[1,2,4]triazole-3-thiol 6

The compound was obtained from 4-amino-3-ethyl-4*H*-[1,2,4]triazole-5-thiol (1 g, 6.9 mmol) and 2,6-difluoro-benzaldehyde (0.98 g, 6.76 mmol) as white powder (0.75 g, 75%) mp: 185–187°C. IR ν_max_ (KBr): 1615 (s), 1245 (m), 875 cm^−1^. ^1^H NMR (CDCl_3_ 400 MHz) δ: 13.8 (s, 1H, S-H), 10.5 (s, 1H, CH = N-), 7.65–7.74 (m, 2H, Ar-H), 7.23–7.34 (t, 1H, Ar-H), 1.23 (t, 3H, -CH_3_), 2.68–2.76 (q, 2H, -CH_2_-). Anal. (C_12_H_11_F_2_N_3_S) C, H, N.

### 4-[(2,6-difluoro-benzylidene)-amino]-5-phenyl-4H-[1,2,4]triazole-3-thiol 7

The product was obtained from 4-amino-3-phenyl-4*H*-[1,2,4]triazole-5-thiol (1 g, 5.2 mmol) and 2,6-difluoro-benzaldehyde (0.74 g, 3.8 mmol) as crystalline solid. Weight: 0.875 g; yield 87%; mp: 195–200°C; IR ν_max_ (KBr): 1612 (s), 1236 (m), 842; ^1^H NMR (CDCl_3_ 400 MHz) δ: 14.35 (s, 1H, S-H), 10.2 (s, 1H, CH = N-), 7.89–7.96 (dd, 2H, Ar-H), 7.66–7.76 (m, 1H, Ar-H), 7.46–7.56 (dd, 2H, Ar-H), 7.28–7.34 (t, 1H, Ar-H); Anal. (C_16_H_11_F_2_N_3_S) C, H, N.

### SPR assay

The gold-coated glass chip (TOYOBO, Osaka, Japan) by immersing in ethanol solution, containing 0.1 mM of the photo-affinity linker and 0.9 mM dummy linker for 12 h as reported previously [26]. The chip was washed successively with ethanol, water, and ethanol. Then the chip is air dried to obtain the photo reactive chip. The compounds 1–9 were spotted on the chip (10 mM), washed, and dried in *vacuo*, and then irradiated at 365 nm under a UV transmission filter (Sigma-Koki, Japan). The compounds were immobilized onto the photo-reactive linker that bound to the gold-surface of the glass chip was set into an SPR imaging instrument (TOYOBO), and each growth factor (50 µg/mL) was injected onto the array surface at 0.1 ml/min and incubated for 10 min. The SPR image and signal data were collected with an SPR analysis program (TOYOBO). The SPR difference image was constructed by using the Scion Image program (Scion, MD).

### Molecular docking study

As a model for docking simulations, we chose the solution structure of the 55-residue heparin-binding domain of VEGF_165_, which has been solved using data from two-dimensional homonuclear and three-dimensional heteronuclear NMR spectroscopy (PDB entry: 2vgh) [31]. We also considered the FGF-2 from the crystal structure of a ternary FGF-FGFR-heparin complex that reveals a dual role for heparin in FGFR binding and dimerization [32]. Molecular docking was carried out using discovery studio program (Accelrys, California, USA). To dock **compound 8** with VEGF_165_, a CDOCKER protocol was used, which is an implementation of the algorithm CDOCKER [33] that allows us to run a refinement docking of any number of ligands with a single protein receptor, which is a grid-based molecular docking method that employs CHARMm. The receptor is held rigid while the ligands are allowed to flex during the refinement. MOLECULAR docking was done by specifying the ligand placement in the active site using a binding site sphere.

### Interaction Analysis

The interaction of VEGF_165_ with heparin in the presence of **compound 8** was examined using a BIAcore J system (BIAcore AB, Uppsala, Sweden). The heparin-immobilized sensor chip was prepared as reported earlier [34]. The interaction of **compound 8** with the heparin binding domain of VEGF_165_ was analysed by injecting the various concentrations of **compound 8** and VEGF_165_ (250 nM) onto the surface of the sensor chip in running buffer, pH 7.4 (HBS-EP; BIAcore AB), containing 10 mM HEPES, 0.15 M NaCl, 3 mM EDTA, and 0.005% (w/v) Tween 20. The flow rate was kept at a moderate speed (30 ml/min) according to the manufacturer's recommendations. Each mixture of the VEGF_165_ and **compound 8** was allowed to interact with the heparin-immobilized sensor chip for 2 min allowing association and dissociation.

### Cell lines

LM8G7 (a gift from M. Miyasaka, Department of Pharmaceutical Chemistry, Osaka University) [23], a highly metastatic murine osteosarcoma cell line with the potential to invade the liver, was cloned from LM8G5 cells [35] as described [28] and cultured in Dulbecco's Modified Eagle's Medium (DMEM) supplemented with 10% (v/v) fetal bovine serum (FBS) (Thermo Trace, Melbourne, Australia), streptomycin (100 µg/mL), penicillin (100 units/mL), 100× non-essential amino acids, b-mercaptoethanol (50 µM), 100× sodium pyruvate, and L-glutamine (2 mM) at 37°C in a humidified 5% CO_2_ atmosphere. The cells were harvested after incubation with 0.1% trypsin/1 mM EDTA in PBS for 5 min at 37°C followed by gentle flushing with a pipette, and subcultured three times a week. Mouse normal vascular endothelial cells (UV♀2) were maintained in DMEM supplemented with 10% (v/v) FBS.

### Proliferation assay

Two tumor cell lines such as LM8, LM8G7, and UV♀2 endothelial were used to evaluate the anti-proliferative activity of the compounds. UV♀2 (1×10^4^ cells) or LM8 or LM8G7 cells (5×10^3^ cells) were seeded in 96-well plate and incubated overnight at 37°C. The tumor cells or endothelial cells stimulated with VEGF (2 ng/ml) were treated with various concentrations of compounds **1–9** for an additional 48 h. 5 µL of TetraColor One reagent was added and incubated for an additional 2–4 h and the absorbance at 450 nm (Bio-Rad) was measured. Further, the effect of **compound 8** on VEGF-mediated proliferation of UV♀2 endothelial was measured. The results were expressed as a percentage of viable cells relative to cells treated with DMSO. The viability of the cells was expressed in percentage terms and IC_50_ value was calculated.

Human umbilical vein endothelial cells (HUVEC) were obtained from the American Type Culture Collection (ATCC). All experiments were done using endothelial cells between passages 3 and 8. HUVECs were maintained in endothelial cell growth medium M200 (Invitrogen) high glucose supplemented with 15% fetal bovine serum, endothelial cell growth supplements (LSGS Medium, Cascade Biologics), and glutamine at 37°C with 5% CO_2_. All cells were maintained as sub confluent cultures and split 1∶3, 24 h before use. HUVECs were seeded on 6-well plates at a density of approximately 1×10^5^ cells/well in M200 medium supplement with LSGS (Low Serum Growth Supplement). The next day cells were stimulated with 10 ng/mL of VEGF in the absence or presence of 1 and 10 µM of **compound 8**. After 48 hrs, AB (Alamar Blue) was added directly into culture media at a final concentration of 10% and the plate was returned to the incubator. Optical density (OD) of the plate was measured at 540 and 630 nm with a standard spectrophotometer at 3–4 h after adding AB. As a negative control, AB was added to medium without cells.

### Real-time proliferation assay

The cell proliferation assay was done using the Real-Time Cell Electronic Sensing (RT-CES) system (ACEA Biosciences, San Diego, CA). LM8G7 (5×10^3^ cells/well) cells were seeded in ACEA's 96× e-plate™ in a final volume of 150 µl [36]. Approximately 24 h after seeding, when in log growth phase, the cells were incubated with 150 µl of DMEM containing various concentrations of **compound 8** (final concentration 2 to 50 µM) or DMEM containing DMSO as control. The effects of **compound 8** on the proliferation of LM8G7 cells were monitored dynamically every 10 min. A cell index (quantitative measurement of cell proliferation) was plotted against time. The IC_50_ values were calculated from concentration-response curves by a non-linear regression analysis using the GraphPad Prism (GraphPad Prism Software Inc., San Diego).

### *In vitro* cell migration and invasion assays

The ability of LM8G7 and UV♀2 cells to migrate and invade was assessed using the BD BioCoat™ chamber with or without Matrigel (BD Biosciences) *in vitro*. The single cell suspensions of LM8G7 cells or UV♀2 cells were prepared by detaching and resuspending in serum-free DMEM. Before the cells were added, the chambers were rehydrated for 2 h in an incubator at 37°C. In the upper chamber (8 µm PET pores), LM8G7 (6×10^4^ cells/ml) or UV♀2 (1×10^5^ cells/ml) cells were added along with **compound 8** (0.5 or 1.0 µM) in 500 µl DMEM and the lower chambers were filled with DMEM containing 10% fetal bovine serum. The Matrigel™ invasion chambers were incubated for a further 22 h at 37°C. After incubation for 24 h, the cells that had migrated or invaded through the membrane alone or the Matrigel-coated membrane remained bound to the underside of the membranes. In some instances, VEGF (2 ng/ml) was added exogenously to understand the effect of **compound 8** on VEGF-induced migration of UV♀2 cells. These cells were stained with Diff-Quik staining kit and counted in five random microscopic fields/filter. The percent inhibition of the invasion or migration of LM8G7 cells or UV♀2 cells by **compound 8** were calculated.

### *In vitro* angiogenesis assay

Matrigel™ (ECM 625, Chemicon) was added to a 24-well plate in a final volume of 100 µL and allowed to solidify at 37°C for 30 min. UV♀2 cells (4×10^5^ cells) were seeded to the ECMatrix™-coated wells in the presence or absence of **compound 8** (0.5 or 1 µM) along with 2 ng/ml VEGF of FGF-2. After 18 h of culture, the reorganization of the sub-confluent monolayer of UV♀2 cells in 3-dimensional ECMatrix™ was monitored and photographed under an Olympus FX380 microscope attached to a 3CCD camera.

### Liver metastasis assay

Nine-week-old female C3H/HeN mice were obtained from Japan SLC (Hamamatsu, Japan) and kept in standard housing. All the experiments were performed according to a protocol approved by the local animal care and use committee of Hokkaido University. C3H/HeN mice were intravenously injected with 1×10^6^ LM8G7 cells in 200 µl of DMEM *via* the tail on day 0. Some mice received an intravenous injection of **compound 8** (0.25, 0.5, or 1.0 mg/kg) suspended in 200 µL of DMEM, on day 3, 5 and 10 after the tumor cell injection. After 4 weeks, the mice were sacrificed and the number of liver nodules was counted macroscopically in the control and **compound 8**-treated animals. Heparin (5 mg/kg) was used as a positive control. The synergetic effects of **compound 8** and heparin were investigated by subsequent administration of **compound 8** (0.25 or 0.5 mg/kg) and heparin (2.5 or 5.0 mg/kg) as described above.

### Ethics statement

All animal experiments were conducted in accordance with protocols specifically approved for this study by the animal care and use committee (IACUC) at Hokkaido University, designed to minimize the numbers of mice used and to minimize any pain or distress.

### Statistical analysis

The statistical analysis was done using software Origin 8 (OriginLab). The Mann-Whitney *U* test was used to determine *P*-values.

## Supporting Information

Figure S1 **Interactions of the compound 8 within the heparin binding pocket of FGF-2 (PDB ID: 1FQ9).** The compound **8** carbons are shown in grey color. The heparin binding domain of FGF-2 amino acids carbons are shown in pink color.
(TIF)

Figure S2 **Effect of Compound 8 on FGF-2-induced tube formation.** The UV♀2 cells were seeded in 6-well plates pre-coated with Matrigel™ (ECM 625, Chemicon) and allowed to solidify the presence or absence of compound 8 (0.5 or 1 µM) at 37°C for 30 min. along with 2 ng/ml FGF-2. After 18 h of culture, the reorganization of the sub-confluent monolayer of UV♀2 cells in 3-dimensional ECMatrix™ was monitored and photographed. The number of intact tubes were counted in five randomly chosen regions and expressed as the percentage of the control, and the results are expressed as mean ± S.D. Inhibition rates of compound 8 on the tube formation of UV♀2 cells was presented.
(TIF)
