# Correction: Anti-Tumor Activity of a Novel HS-Mimetic-Vascular Endothelial Growth Factor Binding Small Molecule

**DOI:** 10.1371/annotation/a685ce21-d7a1-4a5b-8fe8-734a071d53cd

**Published:** 2012-10-25

**Authors:** Basappa, Kazuyuki Sugahara, Kuntebommanahalli N. Thimmaiah, Hemant K. Bid, Peter J. Houghton, Kanchugarakoppal S. Rangappa

Figures 1-8 were placed in the wrong locations.

Figure 8 should be Figure 1 with Figure 1 Legend.

Figure 1 should be Figure 2 with Figure 2 Legend.

Figure 2 should be Figure 3 with Figure 3 Legend.

Figure 3 should be Figure 4 with Figure 4 Legend.

Figure 4 should be Figure 5 with Figure 5 Legend.

Figure 5 should be Figure 6 with Figure 6 Legend.

Figure 6 should be Figure 7 with Figure 7 Legend.

Figure 7 should be Figure 8 with Figure 8 Legend. 

