# Supplementary figures and images for: Anti-Tumor Activity of a Novel HS-Mimetic-Vascular Endothelial Growth Factor Binding Small Molecule
Source: PLoS One. 2012 Aug 15;7(8):e39444. doi: 10.1371/journal.pone.0039444 (PMC3419744; doi:10.1371/journal.pone.0039444)

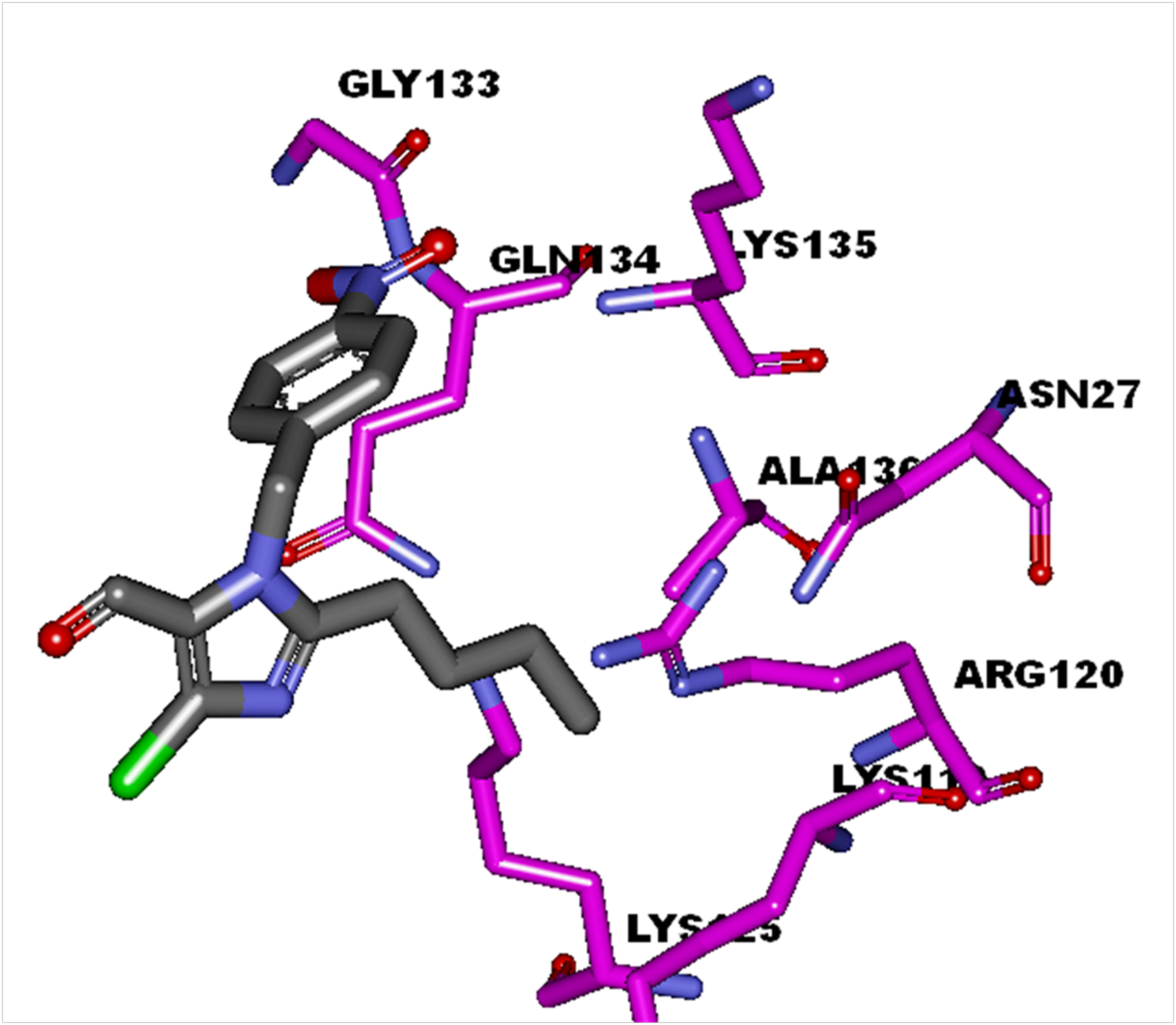

Supplement: Figure S1 — Interactions of the compound 8 within the heparin binding pocket of FGF-2 (PDB ID: 1FQ9). The compound 8 carbons are shown in grey color. The heparin binding domain of FGF-2 amino acids carbons are shown in pink color. (TIF) [file pone.0039444.s001.tif]

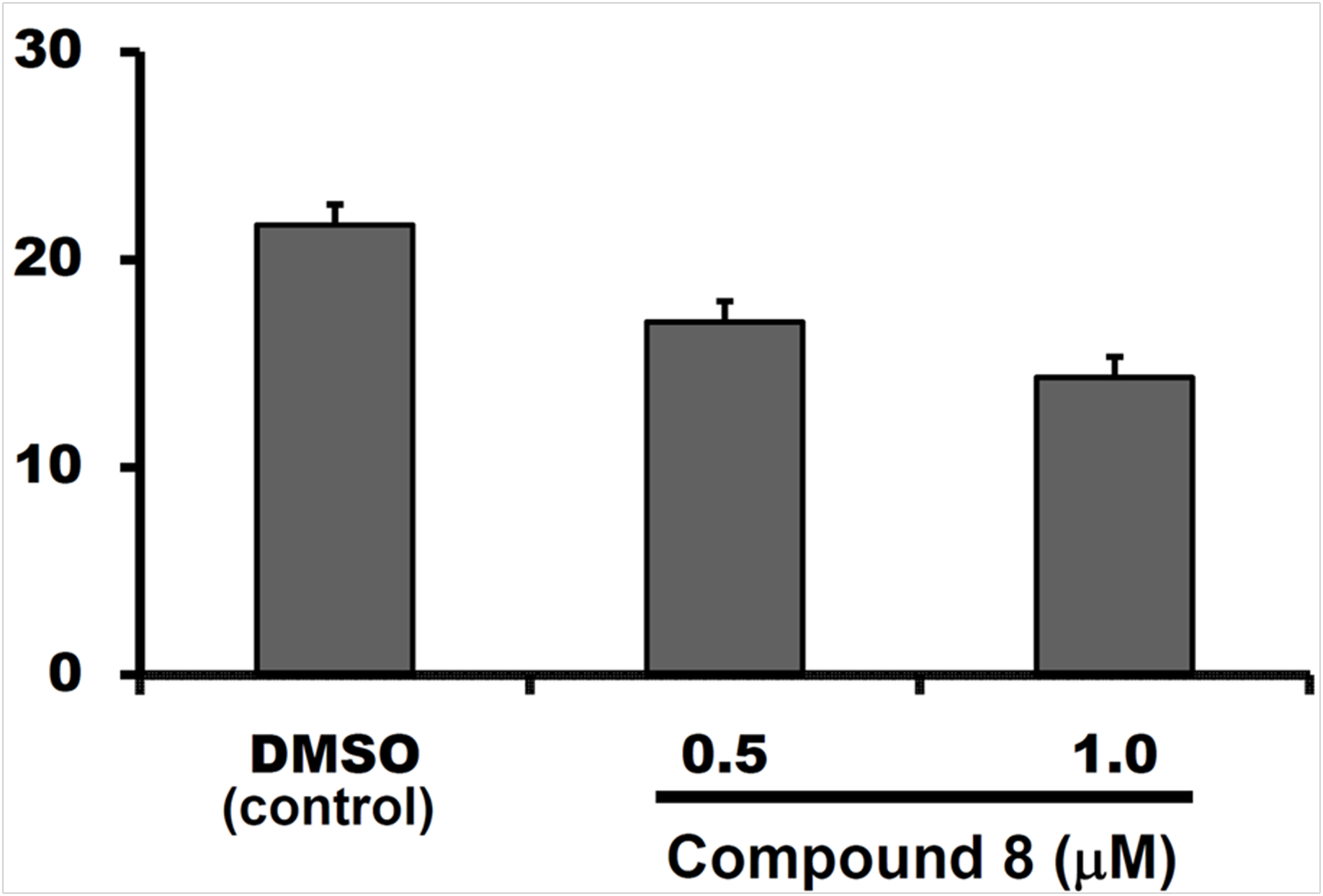

Supplement: Figure S2 — Effect of Compound 8 on FGF-2-induced tube formation. The UV♀2 cells were seeded in 6-well plates pre-coated with Matrigel™ (ECM 625, Chemicon) and allowed to solidify the presence or absence of compound 8 (0.5 or 1 µM) at 37°C for 30 min. along with 2 ng/ml FGF-2. After 18 h of culture, the reorganization of the sub-confluent monolayer of UV♀2 cells in 3-dimensional ECMatrix™ was monitored and photographed. The number of intact tubes were counted in five randomly chosen regions and expressed as the percentage of the control, and the results are expressed as mean ± S.D. Inhibition rates of compound 8 on the tube formation of UV♀2 cells was presented. (TIF) [file pone.0039444.s002.tif]
